# Data collection for outbreak investigations: process for defining a minimal data set using a Delphi approach

**DOI:** 10.1186/s12889-021-12206-5

**Published:** 2021-12-13

**Authors:** Anne Perrocheau, Hannah Brindle, Chrissy Roberts, Srinivas Murthy, Sharmila Shetty, Antonio Isidro Carrion Martin, Michael Marks, Karl Schenkel, the “Minimum Variables for Outbreak Investigation Working Group of the WHO Outbreak Toolkit project”, Anne Perrocheau, Srinivas Murthy, Karl Schenkel, Hannah Brindle, Chrissy Roberts, Michael Marks

**Affiliations:** 1 World Health Organisation, Geneva, Switzerland; 2 London School of Hygiene & Tropical Medicine, London, UK; 3 Centers for Disease Control, Atlanta, USA; 4 Médecins Sans Frontières, London, UK

**Keywords:** Outbreak, Field investigation, Minimum variables, Delphi, Remote settings, Questionnaire

## Abstract

**Background:**

Timely but accurate data collection is needed during health emergencies to inform public health responses. Often, an abundance of data is collected but not used. When outbreaks and other health events occur in remote and complex settings, operatives on the ground are often required to cover multiple tasks whilst working with limited resources. Tools that facilitate the collection of essential data during the early investigations of a potential public health event can support effective public health decision-making. We proposed to define the minimum set of quantitative information to collect whilst using electronic device or not. Here we present the process used to select the minimum information required to describe an outbreak of any cause during its initial stages and occurring in remote settings.

**Methods:**

A working group of epidemiologists took part in two rounds of a Delphi process to categorise the variables to be included in an initial outbreak investigation form. This took place between January–June 2019 using an online survey.

**Results:**

At a threshold of 75 %, consensus was reached for nineteen (23.2%) variables which were all classified as ‘essential’. This increased to twenty-six (31.7%) variables when the threshold was reduced to 60% with all but one variable classified as ‘essential’. Twenty-five of these variables were included in the ‘Time zero initial case investigation’ ‘(T0)’ form which was shared with the members of the Rapid Response Team Knowledge Network for field testing and feedback. The form has been readily available online by WHO since September 2019.

**Conclusion:**

This is the first known Delphi process used to determine the minimum variables needed for an outbreak investigation. The subsequent development of the T0 form should help to improve the efficiency and standardisation of data collection during emergencies and ultimately the quality of the data collected during field investigation.

**Supplementary Information:**

The online version contains supplementary material available at 10.1186/s12889-021-12206-5.

## Background

The rapid investigation of potential public health events^1^ is key to mitigating the harmful effects of these. Operatives on the ground during the early phases of a public health event are often required to cover multiple tasks whilst working with limited resources. When outbreaks and other health events occur in remote and complex settings, these problems can be multiplied. Tools that facilitate the accurate, rapid and efficient collection of essential data during the early investigation of a potential public health event can support effective public health decision-making, whilst adding minimal additional burden to the work of field investigators.

Whilst much attention has been given to the development of analytic tools which aim to facilitate timely analysis and prediction during complex outbreaks, there have been fewer reports which emphasise the need for quality, completeness and timeliness of primary data collection at the field level [1].

At present, there is significant variability in the quality of epidemiological information generated during outbreak investigations. During the 2014–2015 West African Ebola virus disease outbreak, a number of inadequacies in data management were reported, including reports of incomplete case investigation forms and the late arrival of data [2] and the challenges to properly label and classify samples sent to the labs during the investigation. The absence of a central database aimed at linking different sources of data was highlighted as a crucial problem [2] as was the variability of formats from different sources preventing the merger of information and the surfeit of detailed data that were collected but never used in analyses [3]. Studies conducted in South-East Asia and in Europe have also found that a number of data sets collected for outbreak reports were incomplete and/or inaccurate [4, 5]. Combined, the problems of incomplete, delayed, incorrect, excessively detailed and disorganised data sets are the key factors that result in an inability to rapidly assess and report critical indicators on the nature and risks posed by a potential health event. Those observations have been reported for several years and from all over the world.

In resource-limited settings, it is imperative to maximise efficiency by focussing on the primary objectives of data acquisition and to include only variables which may directly inform the immediate response. Alongside this, the methods of data collection and management need to be considered, with particular focus on the need to link multiple data sources and standardise variables for downstream data processing.

In response to the need for standardized data collection instruments for early investigations of public health events, the Health Emergencies Program (WHE) of the World Health Organization (WHO) proposed in 2018 to develop the WHO Outbreak Toolkit (WHO-OT), a novel resource which aims to improve the quality, timeliness and use of data during public health events through a web-based application for field staff. The WHO-OT includes a set of documents providing information about diseases, standardized case definitions, case report forms, laboratory guidelines and standards for data collection [6]. The WHO-OT project adheres to the key epidemiological principles of outbreak investigation, which are to provide an initial description of the situation with regard to the time, place and person. This should allow for 1) an informed assessment of severity and risk of progression of the outbreak, 2) the opportunity to generate hypotheses regarding the potential sources of the hazard(s) and mode of transmission, and 3) to establish whether further laboratory and epidemiological investigations are needed. The WHO-OT project therefore will comprise a set of tools which enable field staff to correctly collect the minimal necessary data for outbreaks (both infectious and non-infectious) of known and unknown cause.

The Delphi process is used to develop consensus amongst a group of experts [7], particularly where there may be little existing evidence for a specific issue [8]. It uses structured group communication through an iterative multistage process [9]. WHO has successfully used the Delphi process in multiple contexts, including recently to define the priority diseases for the WHO Research and Development Blueprint [10].

This paper describes the process used to identify and refine the minimum variables needed during the initial stages of any public health event investigation.

## Methods

In June 2018, WHO convened a working group constituted of 36 experts in epidemiology, clinical medicine, environmental sciences, data management and data sciences who worked in organisations including WHO, the United States Centers for Disease Control (US-CDC), Médecins Sans Frontières (MSF), the European Centre for Disease Prevention and Control (ECDC), the London School of Hygiene & Tropical Medicine (LSHTM), the Helmholtz Centre for Infection Research, and Oxford University. These participants belonged to international organisations involved in field outbreak investigations and were considered experts in working in epidemics in low- and middle-income countries (LMICs). The list of participants was established based on a list of WHO experts in health event investigations, existing working relationships with WHO external partners in data collection, recommendations from experts consulted during recent work for the investigation of outbreaks of unknown origin, and partner institution experts involved in field data collection for all hazards. Terms of references of the working group were circulated and agreed during a first teleconference in June 2018.

The working group was invited to participate in the process of selecting a set of minimum variables (defined as ‘Epi Core Variables’) for event investigations under the WHO-OT project. This process commenced with a review of thirty-one case investigation forms from a range of sources from WHO, US-CDC, MSF, International Federation of Red Cross (IFRC) which had been used in previous health events and included both infectious and non-infectious hazards. From this, the participants selected variables which were common to a generic outbreak situation rather than specific for any pathogen or disease aetiology. These variables, called the “initial list”, contained 82 variables grouped into four categories: 1) Notification interview, 2) Case information, 3) Clinical information and 4) Exposure (Additional file 1). Questions for each variable were phrased in a way that would allow them to be used in any type of health event, whilst also ensuring the data to be comparable across situations. Variables describing clinical signs and symptoms were grouped into two sub-groups including (a) those characterising the severity of any disease and (b) those covering signs and symptoms; Only variables that characterise the severity of any disease, (a), were included in the prioritization exercise.

In order to reach consensus on which of the variables should classified as ‘Epi Core’, the working group decided to use a Delphi process.

Prioritisation of variables was restricted to a sub-group of 26 members of the working group in order to keep a balance among members from different institutions and to restrict the group to experts with regular participation in field outbreak investigations. We conducted the Delphi via an online survey tool in two stages conducted between January 2019 and June 2019 using Enketo web-forms [11]. Delphi participants were sent a guidance document for the process which outlined the purpose of the selection of variables. The variables were ordered by categories and accompanied by a description. This allowed the experts to prioritise the importance of including these as ‘Epi Core variables’ as defined in Table 1. For each variable, this sub-group of participants were asked to determine whether the variable should be retained for inclusion, and if so, to assign its priority in data collection during an outbreak as essential, high, medium, low or unknown (Table 1). The priority classification serves to adapt to the resources available in the field at the time of the outbreak investigation, i.e. if resources are limited, the field officer should collect at a minimum the essential variables. Experts could also provide additional explanatory comments in relation to their categorisations.

**Table 1:** The priority classification and definition of the categorisation of the variables

Category of prioritisation	Definition
Essential	Mandatory variables that should appear on all outbreak investigation forms
High	Variables which are highly desirable however not essential
Medium	Variables which are recommended to be collected when feasible. These should be considered when facing an unusual outbreak that triggers the need for more detailed information, e.g. a novel form of a disease, a changing epidemiological pattern of a known disease, or the suspicion of new risk factors.
Low	Other variables which should be collected when feasible in order to refine the analysis

In January 2019, all 26 members of the sub-group were sent invitations by email to participate in the first round of the Delphi process. Participants were given 55 days to complete the survey. Automated reports of the aggregated results and any additional comments from the first round were generated using R statistical software (version 3.5.3) and were sent to all participants in March 2019, and 25 members of the sub-group were sent invitations to participate in the second round for which the methods were the same. Two additional participants were added to the second round to account for a loss to follow-up of three participants after the first round.

Those variables for which a priority classification reached at least 75% consensus during the first round, were excluded from the second round. In a systematic review of English language Delphi studies, the most common definition for consensus was percentage agreement, with 75% being the median threshold to define consensus [12]. Similar to the first round, 75% was used as the threshold for variables to be included in the second round.

Participants were given 26 days to respond before reports of the results from both the first and second round were disseminated. Following this, all participants were invited to a teleconference to discuss the results, in which 13 attended. Given the limited number of variables which reached a consensus of 75% and the large spread of the results, the group decided to broaden consideration of variables which reached a consensus at 60%. Therefore, we also show those variables which reached at least 60% consensus.

The minimum set of variables served to develop a generic data collection form for outbreak investigation, nominally titled the ‘T0 initial case investigation form’ (“T” for time, “0” for first data collection), to collect the minimum set of data to describe an outbreak of any origin and to build initial hypotheses regarding its origin/source, transmission, aetiology or syndrome. Given that not all of the working group had a clinical background, the Delphi process was only used to facilitate the prioritisation of signs of severity of disease. A sub-group of medical experts then selected pathogen- or syndrome-specific variables from those medical variables included in the initial list of 82 variables (Additional file 1) to be included in the T0 form. WHO laboratory experts were consulted to define variables for laboratory diagnosis to be included in the form.

The T0 form was field tested by members of the National Rapid Response Teams Knowledge Network (RRT KN) in French and English-speaking countries. Feedback was received from three countries, Tunisia, Egypt, Morocco and the Eastern Mediterranean Public Health Network (EMPHNET) who tested the T0 during training of Rapid Response Teams. The field-testing led to the inclusion of additional variables, mainly pertaining to exposure, included in the list as essential in the T0 form following the recommendations issued from field practitioners in the testing process. One variable, ‘neighbourhood/camp/settlement’, was deleted following results of the field-testing.

## Results

Eighty-two variables were selected to be included in the Delphi survey by the sub-group with epidemiological outbreak expertise. Out of 82, nine (11.0%) were categorised as being related to the notification of a case such as the date and location, 31 (37.8%) related to the demographics of the case, 17 (20.7%) to clinical information, and 25 (30.5%) to exposure to the hazard. The list of variables by categories is given in the supplementary data (Additional file 1 ).

Sixteen (61.5%) members of the working group responded to the first-round of the Delphi survey and 17 (68%) to the second round. The distribution of participants and responses by organisational group is shown in Table 2. In both rounds, the majority of participants were from WHO, (61.5 and 60%, respectively).

**Table 2:** Number and Response rates to the first and second round of the Delphi survey by organization

Organisation of participants	Number of participants survey sent to (first round/second round)	Number who responded to the first round and response rate (%)	Number who responded to the second round and response rate (%)
WHO	16 / 15	8 (50.0)	10 (66.7)
Academia	5	4 (80.0)	3 (60.0)
International organisations	5	4 (80.0)	4 (80.0)
**Total**	**26 / 25**	**16 (61.5)**	**17 (68.0)**

Consensus for the classification into the categories was reached for nineteen variables (23.2%) using a threshold of at least 75% (Table 3). All nineteen variables were classified as essential (Tables 4, 5, 6 and 7). On reduction of the threshold for consensus to at least 60%, the number of variables increased to 26 (31.7%) of which 25 variables were classified as essential and one variable was classified as high (Tables 3, 4, 5, 6 and 7). Consensus was highest amongst those related to case information: 35.5% at a threshold of 75% consensus and 45.2% at a threshold of 60% consensus; and lowest for those related to exposure: 8.0% at a threshold of 75% consensus and 12.0% at a threshold of 60% consensus (Table 3).

**Table 3:** The number of variables and the number and percentage of variables which reached consensus by group

Group	Number of variables at the start of the process	Number of variables that reached consensus at 75% in either round (%)	Number of variables that reached consensus at 60% in either round (%)
**Notification interview**	9	2 (22.2)	4 (44.4)
**Case information**	31	11 (35.5)	14 (45.2)
**Clinical information**	17	4 (23.5)	5 (29.4)
**Exposure**	25	2 (8)	3 (12.0)
**Total**	82	19 (23.2)	26 (31.7)

The highest consensus for any variable ranged from 31.3% (*n* = 5) to 100% (*n* = 1) (Tables 4, 5, 6, 7 and Additional file 2).

The tested version of the T0 initial investigation form comprises 43 (52.4%) of the variables from the Delphi process, which included all of those which reached consensus at 75% and all of those which reached consensus at 60% except one indicated above (Tables 4, 5, 6 and 7). In addition to the epidemiologic variables, eleven clinical variables from the initial list were added describing the syndrome and severity of the status of the patient. To those clinical variables, quantitative criteria were also added in the T0 form following recommendations of the medical sub-group of experts. Tables 4, 5, 6 and 7 show that most of the variables selected via the Delphi process relate to the socio-demographic and epidemiology criteria while those related to exposure to agent or mode of transmission were selected during the field testing phase.

**Table 4:** List of variables included in the Delphi process as part of the ‘Notification Interview’ and score of consensus obtained in the two rounds of the survey with final priority category, decision of the medical sub-group and of the field testing results to preserve and final decision to include in the T0

Variable	Highest score (first round) (%)	Highest score (second round) (%)	Final priority category	Medical sub-group	Field testing	Included in the T0 form
Case ID number	100	NA	Essential			X
Date case reported	81.3	NA	Essential			X
Case reported by	43.8	35.3	NA		X	X
Name of reporting facility	68.8	41.2	Essential			X
Interviewer’s identification	31.3	35.3	NA		X	
Interviewer’s organization	31.3	47.1	NA		X	X
Interview date	31.3	64.7	Essential			X
Name of person interviewed (if not the case)	37.5	35.3	NA			
Relation of person interviewed to the case (if not the case)	31.3	35.3	NA			

The highest score refers to the highest percentage given for any priority category. The final category refers to the priority assigned based on reaching consensus at 60%. "X" indicates whether the category was selected by the medical sub-group; included as result of field testing of the T0 form and whether it was included in the final T0 form.

**Table 5:** List of variables included in the Delphi process as part of the ‘Case Information’

Variable	Highest score (first round) (%)	Highest score (second round) (%)	Final priority category	Medical sub-group	Field testing	Included in the T0 form
Surname/Last name	75	NA	Essential			X
First and second names	75	NA	Essential			X
Nickname	37.5	35.3	NA			
Father/mother/guardian (first and last name)	37.5	41.2	NA			
Head of household (first and last name)	50	52.9	NA			
Telephone number	50	47.1	NA		X	X
Date of birth	75	NA	Essential			X
Age at onset (years)	81.3	NA	Essential			X
Age at onset in months	62.5	70.6	Essential			X
Sex	93.8	NA	Essential			X
Nationality	50	41.2	NA			
Ethnic group	43.8	52.9	NA			
Status (refugee, resident, traveler, displaced)	37.5	47.1	NA			
Language spoken at home	37.5	41.2	NA			
Occupation/profession	50	41.2	NA		X	X
If other occupation, specify	31.3	47.1	NA			
Works in health facility	56.3	41.2	NA		X	X
Residential/street address	87.5	NA	Essential			X
Neighborhood/camp/settlement	68.8	58.5	Essential		a	
Landmarks to locate the house	43.8	41.2	NA			
Village/Town/City	93.8	NA	Essential			X
Postcode/ZIP	43.8	41.2	NA			
Administrative level 4 of residence	81.3	NA	Essential			X
Administrative level 3 of residence	75	NA	Essential			X
Administrative level 2 of residence	75	NA	Essential			X
Administrative level 1 of residence	75	NA	Essential			X
Country	68.8	47.1	Essential			X
GPS latitude	50	41.2	NA		X	X
GPS longitude	50	41.2	NA		X	X

^a^indicates that the variable has been withdrawn from the list of epi-core variables after the field testing

**Table 6:** List of variables included in the Delphi process as part of the ‘Clinical Information’

Variable	Highest score (first round) (%)	Highest score (second round) (%)	Final priority category	Medical sub-group	Field testing	Included in the T0 form
Date of illness onset	93.8	NA	Essential			X
Admitted to hospital	75	NA	Essential			X
Outcome of illness	93.8	NA	Essential			X
Duration of symptoms (days)	25	29.4	NA			
Date of death	87.5	NA	Essential			X
Pregnancy	43.8	58.8	NA	X		X
Underlying conditions	31.3	35.3	NA	X		X
Did the patient receive antibiotics prior to admission/specimen collection?	68.8	35.3	High	X		X
Chronic disease	31.3	35.3	NA	X		X
Malnutrition	31.3	41.2	NA	X	X	X
Shock	43.8	47.1	NA	X		X
Intense pain	25	35.3	NA	X		X
Abnormal bleeding	37.5	41.2	NA	X	X	X
Intense fatigue (lethargy) or weakness	31.3	29.4	NA	X		X
Other signs and symptoms, specify	43.8	29.4	NA	X		X
Conscious disorder	43.8	47.1	NA	X	X	X
Shortness of breath	43.8	52.9	NA

**Table 7:** List of variables included in the Delphi process as part of the ‘Exposure’

Variable	Highest score (first round) (%)	Highest score (second round) (%)	Final priority category	Medical sub-group	Field testing	Included in the T0 form
Participation in mass gatherings	56.3	58.8	NA		X	X
If yes, type of mass gathering/s	43.8	35.3	NA		X	X
If yes, locations of mass gatherings	37.5	29.4	NA		X	X
If yes, dates of mass gatherings	37.5	29.4	NA		X	X
Number of household members	37.5	41.2	NA			
Recently or currently sick household members	56.3	NA	NA			
Name of sick household member/s	37.5	41.2	NA			
Relationship with sick household member/s	31.3	35.3	NA			
Sick household member outcomes	31.3	29.4	NA			
Sick household member date of onset	37.5	41.2	NA			
Community members currently sick with a similar illness or were sick with one within the last XX weeks/months?	43.8	41.2	NA			
Other sick community members – names	37.5	41.2	NA			
Relationship with the sick community member	31.3	41.2	NA			
Places of interaction with the community member (e.g. market or church) in the XX weeks/months prior to falling ill	31.3	29.4	NA			
Approximate date of onset of illness for the community member	37.5	41.2	NA			
Outcome of community member’s illness	31.3	29.4	NA			
Did you have any direct contact with any people with similar illness/symptoms in the XX weeks/months prior to the onset of illness?	62.5	52.9	Essential		X	X
Relationship with symptomatic person	37.5	35.3	NA		X	X
Provides the location/s the respondent had contact with the symptomatic person/s	37.5	52.9	NA		X	X
Date of last contact with the person whilst they were symptomatic	43.8	58.8	NA		X	X
Name of symptomatic contact	43.8	47.1	NA			
Have you travelled outside of your current town/village/city sinceXX weeks/months prior to symptom onset?	75	NA	Essential			X
Travel history locations	75	NA	Essential			X
If yes Travel history dates	43.8	35.3	NA			X
If yes Travel history activities	43.8	29.4	NA			

## Discussion

The importance of developing minimum datasets for emerging infectious diseases has been previously described in relation to the design of clinical trials however, this is the first known attempt to determine the core epidemiological information to be collected during outbreaks based on an all-hazards approach [13]. We describe here an approach that allowed a working group from various countries and organisations to participate remotely in a flexible way.

At a threshold of 75%, only 28% of variables included in the Delphi survey reached consensus and even at a threshold of 60% we achieved 39% consensus. Our consensus levels were lower than in a systematic review of one-hundred manuscripts where close to 88% reached consensus [12]. We may have achieved a greater percentage of variables reaching consensus if the categorisation was less specific, such as by limiting it to ‘essential’ and ‘non-essential’ only. Additionally, the background of the working group varied particularly regarding their level of clinical expertise and the pathogens which with they were familiar, which may have influenced some results and potentially resulted in response bias.

The final output of the work is the T0 form, a generic data collection form for outbreak investigations, developed to describe an outbreak and to build hypotheses regarding its origin/source, transmission, cause and/or clinical syndrome. To facilitate data collection in a variety of settings, including areas lacking data connectivity, the T0 has been converted into formats to enable electronic data capture including: KoBoCollect, Go. Data, and the Early Warning, Alert and Response System (EWARS) [14]. In order to standardise the data and to optimise sharing and analysis, a data dictionary for the T0 form was also developed and made available on the website of the Outbreak Toolkit Project**.** Our objective is to stimulate the use of electronic data capture tools in the field.

The T0 form served as the basis for the urgent development of a coronavirus disease 2019 (COVID-19) case based surveillance form that was shared with all countries and which allowed to rapidly collect the minimum information needed to monitor the COVID-19 pandemic [15, 16]. The variables of the T0 form have also served as the backbone for the development of a Time 1 (T1) questionnaire for investigation of unknown diseases, with a greater focus on clinical symptoms and laboratory results and which is available on demand. The development of the T1 questionnaire was conducted separately to the Delphi process.

We hope that this first attempt to develop a standard questionnaire to be used at the start of any outbreak investigation will continuously be revised by those using it. Also the principle of a generic questionnaire, developed as a tool to support field epidemiologists, implies that it should be adapted to the specific outbreak context at the start of the investigation.

## Conclusions

This is the first reported systematic Delphi process which aimed to define a common set of core variables to be included in an initial case investigation form for outbreak investigations. This tool will support the global standardisation of data collection during the early stage of public health events, allowing for improved efficiency and timeliness of data capture in challenging settings.

## Supplementary Information

**Additional file 1.**  Selected Variables and Definitions by Category.

**Additional file 2: Fig. S1.** The percentage of participants who categorised each variable as essential, high, medium, low or unknown (‘nil’). The red bars refer to the responses from the first round and the blue bars, the second. A line is given at the 75% threshold used to determine consensus following the first round.

## Data Availability

The datasets used and/or analysed during the current study are available from the corresponding author on reasonable request.

## Abbreviations

ECDC: The European Centre for Disease Prevention and Control
EMPHNET: Eastern Mediterranean Public Health Network
EWARS: Early Warning, Alert and Response System
IFRC: International Federation of the Red Cross
LMICs: Low- and middle-income countries
LSHTM: The London School of Hygiene & Tropical Medicine
MSF: Médecins Sans Frontières
RRT: Rapid Response Team
US-CDC: The United States Centers for Disease Control
WHO-OT: World Health Organization Outbreak Toolkit
WHO: World Health Organization

## Footnotes

1 A public health event is any event that may have negative consequences for human health. The term includes events that have not yet led to disease in humans but have the potential to cause human disease through exposure to infected or contaminated food, water, animals, manufactured products or environments. In contrast, an outbreak denotes that the event has already caused disease (WHO definition).

## References

[1] Polonsky JA, Baidjoe A, Kamvar ZN, Cori A, Durski K, Edmunds WJ (2019). Outbreak analytics: a developing data science for informing the response to emerging pathogens. Philos Trans R Soc Lond Ser B Biol Sci.

[2] Owada K, Eckmanns T, Kamara KB, Olu OO (2016). Epidemiological data management during an outbreak of Ebola virus disease: key issues and observations from Sierra Leone. Front Public Health.

[3] Cori A, Donnelly CA, Dorigatti I, Ferguson NM, Fraser C, Garske T (2017). Key data for outbreak evaluation: building on the Ebola experience. Philos Trans R Soc Lond B Biol Sci.

[4] Lawpoolsri S, Kaewkungwal J, Khamsiriwatchara A, Sovann L, Sreng B, Phommasack B (2018). Data quality and timeliness of outbreak reporting system among countries in greater Mekong subregion: challenges for international data sharing. PLoS Negl Trop Dis.

[5] Kroneman A, Harris J, Vennema H, Duizer E, van Duynhoven Y, Gray J (2008). Data quality of 5 years of central norovirus outbreak reporting in the European network for food-borne viruses. J Public Health (Oxf).

[6] World Health Organization (2020). WHO Outbreak Toolkit.

[7] Dalkey N, Helmer O (1963). An experimental application of the Delphi method to the use of experts. Manag Sci.

[8] Thangarathium S, Redman CWE (2005). The Delphi technique. Obstet Gynaecol.

[9] Okoli C, Pawlowski SD (2004). The Delphi method as a research tool: an example, design considerations and applications. Inf Manag.

[10] Mehand MS, Millett P, Al-Shorbaji F, Roth C, Kieny MP, Murgue B (2018). World Health Organization Methodology to Prioritize Emerging Infectious Diseases in Need of Research and Development. Emerg Infect Dis.

[11] Enketo. 2020 [Available from: https://enketo.org/].

[12] Diamond IR, Grant RC, Feldman BM, Pencharz PB, Ling SC, Moore AM (2014). Defining consensus: a systematic review recommends methodologic criteria for reporting of Delphi studies. J Clin Epidemiol.

[13] Rojek AM, Moran J, Horby PW (2020). Core minimal datasets to advance clinical research for priority epidemic diseases. Clin Infect Dis.

[14] T0 Initial case investigation form [Available at: https://www.who.int/emergencies/outbreak-toolkit/data-collection-standards/t0-initial-case-investigation-form

[15] Interim case reporting form for 2019 Novel Coronavirus (2019-nCoV) of confirmed and probable cases: WHO minimum data set report form, 21 January 2020: available at https://apps.who.int/iris/handle/10665/332411

[16] World Health Organization. Case-based reporting form 2020 [Available from: https://www.who.int/publications/i/item/case-based-reporting-form.

