# Supplementary material for: Data collection for outbreak investigations: process for defining a minimal data set using a Delphi approach
Source: BMC Public Health. 2021 Dec 13;21:2269. doi: 10.1186/s12889-021-12206-5 (PMC8666343; doi:10.1186/s12889-021-12206-5)
Supplement: Supplementary file 1 — Additional file 1. Selected Variables and Definitions by Category. [file 12889_2021_12206_MOESM1_ESM.docx]

**Additional file 1: Selected Variables and Definitions by Category**

| **Number** | **Category** | **Label of variable** | **Definition of variable** |
| --- | --- | --- | --- |
| 1 | Interview | Case ID number: | Unique case identification number. |
| 2 | Interview | Date case reported (DD.MM.YYYY): | Date the case was first reported (notified) to public health authorities. |
| 3 | Interview | Case reported by: | Type of facility that reported/notified the case. Note, categories should be adapted to suite local context. |
| 4 | Interview | Name of reporting facility: | Name/number/ID code of facility that reported the case. |
| 5 | Interview | Interviewer's identification | Name or identification code/number of person conducting the interview and completing this form. |
| 6 | Interview | Interviewer's organization: | Unit/Department/Organization that interviewer is representing. Note, label should be adapted to suite local context. |
| 7 | Interview | Interview date (DD.MM.YYYY): | Date the case was interviewed. |
| 8 | Interview | Name of person interviewed (if not the case): | Name of the person being interviewed (if not the case). |
| 9 | Interview | Relation of person interviewed to the case (if not the case): | Relationship of the person being interviewed to the case |
| 10 | Case information | Surname/Lastname: | Case's surname. |
| 11 | Case information | First and second names | Case's first and second names. |
| 12 | Case information | Nickname: | Case's nickname, familiar name or name by which he/she would like to be addressed. |
| 13 | Case information | Father/mother/guardian (first and last name): | Name of father, mother or legal guardian of child/minor case. |
| 14 | Case information | Head of household (first and last name): | Name of head of household of ordinary residence. Used to recontact the patient or his/her contacts (operationality) |
| 15 | Case information | Tel number | telephone number of the patient or of the close parent |
| 16 | Case information | Date of birth (DD.MM.YYYY): | Date that the case was born on. |
| 17 | Case information | Age at onset (years): | Conditional: if birth date not available, ask the age in years on the date of illness onset. |
| 18 | Case information | Age at onset in months | Conditional : For children less than 5 years. For vaccine preventable disease . |
| 19 | Case information | Sex: | Biological sex. That is the biological differential characteristics (chromosomes, hormonal profiles, internal and external sex organs) that best describes the case. |
| 20 | Case information | Nationality: | The nation/country the case identifies as belonging to, whether by birth or naturalization. |
| 21 | Case information | Ethnic group: | The ethnic group the case identified as belonging to. An ethnic group/ethnicity refers to a category of people who identify with each other based on similarities such as common ancestry, language, society, culture or nation. |
| 22 | Case information | Status | Administrative status as member of the host population, displaced, refugee, migrant, traveler |
| 23 | Case information | Language spoken at home: | Primary language spoken in the case's household. |
| 24 | Case information | Occupation/profession: | Primary occupation, profession, type of employment, vocation include voluntary/unpaid work and studies. Categories to be pre-defined based on known risk factors for disease. |
| 25 | Case information | If other occupation, specify: | If other, specific occupation. |
| 26 | Case information | Works in health facility: | Does the case work/volunteer in any type of health facility (e.g. mobile unit, clinic, community health centre, hospital, etc.)? Includes all professions/positions/activities involving a health facility. |
| 27 | Case information | → If yes, list facility(ies): | Name(s) of health facility(ies) where the case works/volunteers. |
| 28 | Case information | → If yes, functions/position: | Primary or usual functions/position/duties/activities at the health facility where the case works/volunteers. Note, this is distinct from prior questions on occupation/profession. |
| 29 | Case information | Residential/street address: | Address (household number/identifiers, street name, block, etc.) where the case currently/usually resides. |
| 30 | Case information | Neighbourhood/camp/settlement: | Neighbourhood/camp/settlement/suburb where the case currently/usually resides. |
| 31 | Case information | Landmarks to locate the house: | Landmarks, easy to recognize object or feature of a landscape or town that enables one establish a location of the case's current/usual place of residence. |
| 32 | Case information | Village/Town/City: | Town/city where the case currently/usually resides. |
| 33 | Case information | Postcode/ZIP: | Postal code or ZIP code are where the case currently/usually resides. |
| 34 | Case information | [User defined] | Administrative level 4: Forth sub-national level boundary (e.g. community, ward, parish, commune, etc.) within which the case currently/usually resides. |
| 35 | Case information | [User defined] | Administrative level 3: Third sub-national level boundary (e.g. sub-district, commune, zone, municipality, etc.) within which the case's currently/usually resides. |
| 36 | Case information | [User defined] | Administrative level 2: Second sub-national level boundary (e.g. district, county, local government area, department, province, municipality, etc.) within which the case's currently/usually resides. |
| 37 | Case information | [User defined] | Administrative level 1: First sub-national boundary (e.g. province, state, territory prefecture, region, etc.) within which the case's currently/usually resides. |
| 38 | Case information | Country: | Administrative level 0: Country/territory/national boundary within which the case currently/usually resides. If transborders or international spread of outbreak |
| 39 | Case information | GPS latitude: | Latitude where the case currently/usually resides, preferably expressed as decimal degrees. Latitude is the geographic coordinates that specifies the north-south position of a point on the Earth's surface, usually obtained using a global position system (GPS) enabled device (e.g. smart phone) or a web mapping application (e.g. Open Street Map, Google Maps, etc.). |
| 40 | Case information | GPS longitude: | Longitude where the case currently/usually resides, preferably expressed as decimal degrees. Longitude is the geographic coordinates that specifies the east-west position of a point on the Earth's surface, usually obtained using a global position system (GPS) enabled device (e.g. smart phone) or a web mapping application (e.g. Open Street Map, Google Maps, etc.). |
| 41 | Clinical information | Date of illness onset (DD.MM.YYYY) | Date of first appearance of the signs or symptoms of the illness/disease. |
| 42 | Clinical information | Admitted to hospital: | Was the case hospitalized, admitted to a hospital or other health facility as an inpatient? |
| 43 | Clinical information | Outcome of illness: | Outcome of illness. Where possible, verify status after a suitable period of time [user defined] and update. |
| 44 | Clinical information | Duration of symptoms (days): | Number of days from symptom onset to resolution of illness (including deaths). Write "ongoing" or "N/A" if symptoms/sequale are ongoing. |
| 45 | Clinical information | → If died, date of death (DD/MM/YYYY): | If the case died, date of death. |
| 46 | Clinical information | Pregnancy | Is the patient pregnant? |
| 47 | Clinical information | Underlying conditions | Does the patient have co-morbidity (ies) existing prior to admission that is (are) active problems? |
| 48 | Clinical information | Did the patient received antibiotic prior to admission/specimen collection? |  |
| 49 | Clinical information | Chronic disease | Is the patient suffering from a chronic disease? If yes indicate name |
| 50 | Clinical information | Malnutrition | if possible provide the MUAC score or any measure that help evaluating the severity of malnutrition |
| 51 | Clinical information | Shock | Septic, haemorrhagic or cardiogenic shock  ISARIC definition for shock : Shock may be due to sepsis, intravascular volume depletion (including fluid loss and/or blood loss), myocardial dysfunction, or vasodilatation leading to poor end-organ perfusion. Cardinal features of shock include persistent hypotension, oliguria/anuria, prolonged capillary refill time, altered mental state, metabolic acidosis and sometimes cool and clammy skin. Only some of these features may be present. Hypotension may be absolute (systolic BP <90mmHg) or relative (a decrease in systolic BP >40mmHg) |
| 52 | Clinical information | Intense pain | Indicate site(s) of intense pain: headaches (pain anywhere in the region of the head or neck), myalgia (abnormal pain in the muscles), arthralgia (abnormal pain in one or more joints), back pain, chest pain, general pain, other site |
| 53 | Clinical information | Abnormal bleeding | Any recent abnormal bleeding from any site that the patient reports |
| 54 | Clinical information | Intense fatigue (lethargy) or weakness: | Intense fatigue, an abnormal state of drowsiness (lethargy or feeling lethargic). |
| 55 | Clinical information | Other signs and symptoms, specify: | Other symptoms, specify: |
| 56 | Clinical information | Conscious disorder: | Presence of any conscious disorder |
| 57 | Clinical information | Shortness of breath | Difficulty breathing (dyspnoea), abnormal shortness of breath or intense tightening of the chest while resting or during light physical activity (e.g. normal walking), noisy respiration |
| 58 | Exposure | Participation in mass gatherings | Indicates if the repondent attended any mass gatherings (e.g. funerals, festivals, sports events etc) in the XX weeks/months prior to symptom onset |
| 59 | Exposure | If yes, type of mass gathering/s | Provides a list of mass gatherings the respondent attended in the XX weeks/months prior to symtom onset. |
| 60 | Exposure | If yes, locations of mass gatherings | Provides locations for the list of mass gatherings the respondent provided in the answer to the above question |
| 61 | Exposure | If yes, dates of mass gatherings | Provides dates for the list of mass gatherings the respondent provided in the answer to the above question |
| 62 | Exposure | No. household members | Respondent's number of household members (definition of household to be locally decided - might need to be set to total number for the month) |
| 63 | Exposure | Recently or currently sick household members | Indicates if there are any other household members currently ill with a similar illness or sick with a similar illness in the last XX weeks/months |
| 64 | Exposure | Name of sick household member/s | Provides a list of names of currently or recently sick household members |
| 65 | Exposure | Relationship with sick household member/s | Indicates the relationship between the respondent and the sick household member/s (1=parent, 2=child, 3=non-relative, 4=partner etc) |
| 66 | Exposure | Sick household member outcomes | Indicates the outcome of illness for listed sick household members (1=recovered, 2=ongoing, 3=died of illness, 4=died of other cause, 5=sequale, 6=unknown) |
| 67 | Exposure | Sick household member date of onset | Approximate date of onset of the household member's illness |
| 68 | Exposure | Community members currently sick with a similar illness or were sick with one within the last XX weeks/months? | Indicates if the respondent aware of any other community members (not listed as a household member) who are currently sick with a similar illness or were sick with one recently. |
| 69 | Exposure | Other sick community members - names | Name of the sick community member (if known and willing to disclose) |
| 70 | Exposure | Relationship with the sick community member | relationship with the sick community member (e.g 1=friend, 2=workmate, 3=relative, 4=romantic partner etc.) |
| 71 | Exposure | Places of interaction with the community member (e..g market or church) in the XX weeks/months prior to falling ill | Places that the respondent most commonly meets or congregates with the community member (if many places or they have nothing in common at all just write that). If there is a specific thing like "swimming lessons", school etc. write that. |
| 72 | Exposure | Approximate date of onset of illness for the community member | Approximate date of onset of illness for the community member |
| 73 | Exposure | Outcome of community member's illness | Indicates the outcome of illness for listed sick household members (1=recovered, 2=ongoing, 3=died of illness, 4=died of other cause, 5=sequale, 6=unknown) |
| 74 | Exposure | Did you have any direct contact with any people with a similar illness/symptoms in the XX weeks/months prior to the onset of illness. | Indicates if the respondent had any direct contact with any symptomatic people with similar symptoms in the XX weeks/months prior to the onset of illness. |
| 75 | Exposure | Relationship with symptomatic person | relationship with the sick community member (e.g 1=friend, 2=workmate, 3=relative, 4=romantic partner etc.) |
| 76 | Exposure | Provides the location/s the respondent had contact with the symptomatic person/s | Provides the location/s the respondent had contact with the symptomatic person/s |
| 77 | Exposure | Date of last contact with the person whilst they were symptomatic | Approximate date of last interaction with the person whilst they were still symptomatic (if dead, date of last contact with the body) |
| 78 | Exposure | Name of symptomatic contact | Provides the name of the symptomatic person the respondent had contact with |
| 79 | Exposure | Have you travelled outside of your current town/village/city since Xx week/months prior to symptom onset | Indicates if the respondent has travelled outside of the current IDP/town/village/city since XX weeks/months prior to their symptom onset. |
| 80 | Exposure | Travel history locations | Provides a list of locations the respondent travelled to in XX weeks/months prior to symptom onset |
| 81 | Exposure | Travel history dates | Provides dates to accompany the list of locations the respondent provided in the answer to the question above |
| 82 | Exposure | Travel history activities | Provides a list of key activities undertaken by the respondent at the locations listed (e.g attended a market) |
