# Supplementary material for: Data collection for outbreak investigations: process for defining a minimal data set using a Delphi approach
Source: BMC Public Health. 2021 Dec 13;21:2269. doi: 10.1186/s12889-021-12206-5 (PMC8666343; doi:10.1186/s12889-021-12206-5)
Supplement: Supplementary file 2 — Additional file 2: Fig. S1. The percentage of participants who categorised each variable as essential, high, medium, low or unknown (‘nil’). The red bars refer to the responses from the first round and the blue bars, the second. A line is given at the 75% threshold used to determine consensus following the first round. [file 12889_2021_12206_MOESM2_ESM.docx]

**Additional file 2**

**Figure S1 The percentage of participants who categorised each variable as essential, high, medium, low or unknown (‘nil’). The red bars refer to the responses from the first round and the blue bars, the second. A line is given at the 75% threshold used to determine consensus following the first round.**
